# Socioeconomic Inequalities in Smoking and Smoking Cessation Due to a Smoking Ban: General Population-Based Cross-Sectional Study in Luxembourg

**DOI:** 10.1371/journal.pone.0153966

**Published:** 2016-04-21

**Authors:** Anastase Tchicaya, Nathalie Lorentz, Stefaan Demarest

**Affiliations:** 1 Luxembourg Institute of Socio-Economic Research (LISER), Department of Living Conditions, Health Research Team, Esch-sur-Alzette, Luxembourg; 2 Scientific Institute of Public Health (WIV-ISP), OD Public Health and Surveillance, Brussels, Belgium; Universität Bochum, GERMANY

## Abstract

This study aimed to measure changes in socioeconomic inequalities in smoking and smoking cessation due to the 2006 smoking ban in Luxembourg. Data were derived from the PSELL3/EU-SILC (Panel Socio-Economique Liewen Zu Letzebuerg/European Union—Statistic on Income and Living Conditions) survey, which was a representative survey of the general population aged ≥16 years conducted in Luxembourg in 2005, 2007, and 2008. Smoking prevalence and smoking cessation due to the 2006 smoking ban were used as the main smoking outcomes. Two inequality measures were calculated to assess the magnitude and temporal trends of socioeconomic inequalities in smoking: the prevalence ratio and the disparity index. Smoking cessation due to the smoking ban was considered as a positive outcome. Three multiple logistic regression models were used to assess social inequalities in smoking cessation due to the 2006 smoking ban. Education level, income, and employment status served as proxies for socioeconomic status. The prevalence of smoking decreased by 22.5% between 2005 and 2008 (from 23.1% in 2005 to 17.9% in 2008), but socioeconomic inequalities in smoking persisted. Smoking prevalence decreased by 24.2% and 20.2% in men and women, respectively; this difference was not statistically significant. Smoking cessation in daily smokers due to the 2006 smoking ban was associated with education level, employment status, and income, with higher percentages of quitters among those with a lower socioeconomic status. The decrease in smoking prevalence after the 2006 law was also associated with a reduction in socioeconomic inequalities, including differences in education level, income, and employment status. Although the smoking ban contributed to a reduction of such inequalities, they still persist, indicating the need for a more targeted approach of smoke-free policies directed toward lower socioeconomic groups.

## Introduction

Smoking is a major cause of premature mortality and morbidity and is a global public health issue. With the adoption of smoke-free policies in most developed countries, the general prevalence of smoking has declined, but rates remain particularly high among lower socioeconomic groups, including those with lower education levels, incomes, and employment status [1–8].

The relatively high smoking prevalence in the lowest socioeconomic groups can partly explain the socioeconomic inequalities in health in most developed countries [9–12], where socioeconomic inequalities in smoking contribute to socioeconomic inequalities in mortality [9,10,13,14]. In many European countries, approximately 30% and 15% of socioeconomic inequalities in mortality and morbidity, respectively, among men are attributable to smoking [12]. However, only a few studies have addressed socioeconomic inequalities in smoking or the relationship between socioeconomic inequalities in smoking and health disparities in Luxembourg.

The Luxembourg Cancer Foundation reported that 22% of the population aged ≥15 years were smokers in 2011, following a peak prevalence of 33% in 2003 [15]. Among smokers, 58% wanted to stop, 15% wanted to reduce their consumption of tobacco, and 26% did not want to change anything [15]. To address the harmful effects of smoking on population health, the Government of the Grand Duchy of Luxembourg adopted a smoke-free law on September 5, 2006 that was intended to "protect everyone against the harmful effects of passive smoking" and was aimed at "restricting advertising of tobacco and its products, banning smoking in certain places and prohibition of placing on the market of tobacco for oral use" [16]. Specifically, these places included public areas, such as restaurants, bars, hospitals, schools, museums, theatres, modes of public transportation (trains, buses, and airplanes), nursing homes and accommodations for the elderly, and the workplace. Other measures, including fiscal measures and control of tobacco prices in Luxembourg, had little impact on most smokers, including those residing in neighbouring countries such as Belgium, France, and Germany. Indeed, many smokers in these countries often travelled to Luxembourg to purchase cigarettes because of the comparatively better average prices for a pack of 20 cigarettes: € 5.0 in Luxembourg, € 5.47 in Germany, € 5.79 in Belgium, € 7.0 in France, € 10.0 in Ireland, and € 11.0 in Great Britain in 2014 [17]. As a consequence, tobacco consumption volumes per capita in Luxembourg are artificially high and do not reflect the actual consumption of tobacco by its residents.

In Europe, several recent studies in the general population have shown a continuous decline in the smoking prevalence and a corresponding increase in the smoking cessation rate. Smoking cessation has also been associated with socioeconomic position [8,18–21]; more educated smokers are more likely to quit smoking than are less educated smokers [8,19,20], and income and employment status can predict smoking cessation in the general population [22–25]. Analysing the socioeconomic determinants of smoking cessation helps to explain how policies aiming to reduce smoking are translated in terms of distribution and social benefits among different socioeconomic groups.

Changes in smoking prevalence and smoking cessation in Luxembourg have not been studied. The present study aimed to measure the extent of socioeconomic inequalities in smoking and smoking cessation in the general population in Luxembourg and to assess if the smoking ban had a positive effect on smoking cessation, regardless of socioeconomic status.

## Materials and Methods

### Data and data sources

Data were derived from the PSELL3/EU-SILC (Panel Socio-Economique Liewen Zu Letzebuerg/European Union—Statistic on Income and Living Conditions) survey on income and living conditions of households conducted in Luxembourg in 2005, 2007, and 2008. The cross-sectional pooled data of the successive surveys allows researchers to track changes in income and living conditions of households for use in social protection policies [26]. The PSELL3/EU-SILC survey was a longitudinal survey of approximately 8,000 people aged ≥16 years (7,535 in 2005, 7,913 in 2007, and 7,638 in 2008). Only the cross-sectional data from the surveys were used for the present study. People residing in institutions, such as hospitals, retirement homes, nursing homes, or long-term care facilities, were not eligible for interview.

### Ethics statements

All of the households that were selected for participation in the PSELL/EU-SILC survey received a pre-notification letter in which the survey topic and contents, interview, and voluntary nature of the study were described. Therefore, signed informed consent was not necessary. The consent of children to participate was implicit and based on the participation of their elders.

The survey design and questionnaires were approved by the National Commission for Data Protection. This study is part of the Monitoring and Dynamics of Health status through Risk Factors for Cardiovascular disease project (MDYNRFC), funded by the National Research Fund. The MDYNFRC-project was approved by both the National Research Fund and the National Commission for Data Protection.

### Smoking prevalence

Smoking prevalence was calculated based on the question “Do you smoke?”, with “Yes, every day”, “Yes, sometimes”, and “No” as possible answers. For the purpose of this study, the first two “Yes” responses were combined into one. Therefore, the prevalence of smoking was defined as the proportion of people who responded that they smoked daily or sometimes.

### Smoking cessation due to the 2006 smoke-free law

In general, the smoking cessation rate is defined as the number of former smokers who quit smoking divided by the number of ever smokers and multiplied by 100% [23]. In the present study, we defined the proportion of smokers who ceased smoking as the number of smokers who quit smoking after the introduction of the 2006 smoke-free law divided by the number of smokers who smoked daily at the onset of this law and multiplied by 100%. This was based on the PSELL3/EU-SILC survey question “Did you stop smoking because of the smoke-free law concerning the prohibition to smoke in public areas (restaurants, bars, hospitals, schools, museums, theatres, modes of public transportation, nursing homes and accommodation for the elderly, and the workplace)?”, which was only asked of the people who smoked daily. Possible responses were “Yes, that has been decisive”, “No, but that has certainly played a role”, and “No, I stopped smoking before the smoke-free law”. Because only a small number of people decided to quit smoking after the 2006 smoke-free law, the first two responses were combined for analyses, which allowed measurement of the direct and indirect (even partial) influence of the 2006 smoke-free law on daily smoking. A sensitivity analysis using a receiver operating characteristic curve that was conducted for the first response category alone (area under the curve, 0.6082) or in combination with the second response category (area under the curve, 0.6290) showed a low risk of misclassifying individuals.

### Socio-demographic variables

Socio-demographic variables were age, sex, and marital status. Age was defined as a categorical variable. Four categories of marital status were used: single, married, divorced/separated, or widowed.

### Socioeconomic variables

Education level, living standards, and employment status were used as indicators of socioeconomic status. Education level was defined as the highest level of education based on the International Standard Classification of Education adopted by the UNESCO [27] and categorised into primary, secondary, or tertiary education. Household equivalent income was calculated as household income divided by the equivalent number of household consumption units. Equivalent income was defined using a commonly used quartile structure: the first quartile included individuals belonging to the 25% of households with the lowest equivalent income, while the 4th quartile included those belonging to the 25% of households with the highest equivalent income. Because the aim of the PSELL/EU-SILC survey was to assess the income situation of the population, household income data were of utmost importance. For missing values, the survey managers imputed income.

Employment status was defined as employed, self-employed, unemployed, retired/disabled, student or apprentice, or others.

### Statistical analysis

A descriptive analysis was conducted to measure the prevalence of smoking and the change in prevalence between 2005 and 2008 for each sex in terms of the following socioeconomic factors: education level, household equivalent income, and employment status. The change in smoking prevalence was calculated in relative terms as follows: percentage of change in smoking prevalence = ((prevalence in 2008 –prevalence in 2005)/prevalence in 2005) × 100. The trend (p-value) was assessed using the Cochran-Armitage trend test.

Measures of inequality, such as the smoking prevalence ratio and the disparity index, were used to calculate the magnitude and temporal trends of socioeconomic inequalities in smoking for each sex. The smoking prevalence ratio was defined as the smoking prevalence for people with the lowest socioeconomic status divided by the smoking prevalence for people with the highest socioeconomic status. In addition, the smoking prevalence ratio was adjusted by age using a log-binomial model. Typically, the prevalence ratio for education level is equal to the smoking prevalence for people with a primary education divided by the smoking prevalence for people with a tertiary education. The disparity index measures “the mean deviation of the group rates from some reference point (usually the best group rate) as a proportion of that reference point” [28–29]. The disparity index expresses the summed differences as a proportion of the reference rate. The total prevalence of smoking was used as the reference rate for each social group [29]. Thus, the disparity index was expressed as a percentage of the total smoking prevalence. Analysis of the distribution of people who quit smoking by socioeconomic and demographic characteristics was performed with the chi-square test (p-value) to determine if there was a significant difference between the categories.

Logistic regression models, adjusted for age and sex, were used to determine the odds ratio (OR) for quitting smoking following the 2006 smoking ban based on socioeconomic factors [30]. This analysis was restricted to people who were former smokers in 2007 (n = 1,804) that stopped smoking (at least partially) due to the 2006 smoking ban. Three models were considered: (i) education level as the socioeconomic factor; (ii) household equivalent income as the socioeconomic factor; and (iii) employment status as the socioeconomic factor. Given the small number of cases who quit smoking, the analyses were not stratified by sex, but sex was included as a confounding factor. Correlations and multicollinearity among the three socioeconomic factors were analysed; the correlation coefficients were <0.45, and multicollinearity was almost absent.

All results were weighted relative to the sample size. All statistical analyses were performed using SAS 9.4 software (SAS Institute Inc., Cary, NC, USA).

## Results

In relative terms, the overall smoking prevalence in Luxembourg significantly decreased by 22.5%, from 23.1% in 2005 to 17.9% in 2008 (p < 0.0001; Table 1). The prevalence of smoking significantly decreased by 24.2% and 20.2% in men and women, respectively (both p < 0.0001; Tables 2 and 3). The trend for the decrease in men was significant for all age groups, but was most marked in men aged 35–49 years (-27.4%; p < 0.0001) and ≥65 years (-49.5%; p < 0.0001). The trend for the decrease in women was only significant for those aged 16–24 years (-41.9%; p < 0.0001) and 35–49 years (-24.1%; p = 0.0001). Regarding marital status, the decrease was most marked in widowed men (-45.4%), married men (-29.0%), and divorced/separated women (-23.6%).

**Table 1:** Change in the overall smoking prevalence in Luxembourg from 2005 to 2008.

	2005 (n = 7,535)	2007 (n = 7,913)	2008 (n = 7,638)	Change, 2005 to 2008, % [95% CI]	p*
**All**	23.1	18.9	17.9	-22.5 [-27.3; -17.4]	<0.0001
**Age (years)**					
16–24	29.1	23.5	20.9	-28.2 [-38.5; -16.1]	<0.0001
25–34	26.8	21.4	23.7	-11.8 [-22.8; 0.7]	0.0095
35–49	26.3	21.4	19.5	-25.9 [-33.3; -17.6]	<0.0001
50–64	21.0	18.5	17.4	-17.3 [-28.2; -4.8]	0.0033
≥65	11.0	9.1	7.5	-32.3 [-47.1; -13.4]	0.0010
**Marital status**					
Never married	28.9	24.0	23.6	-18.4 [-26.3; -9.7]	<0.0001
Married	19.1	15.3	14.1	-26.2 [-32.9; -18.9]	<0.0001
Divorced/Separated	41.1	34.4	31.1	-24.3 [-35.4; -11.3]	0.0002
Widowed	14.8	13.0	12.3	-16.8 [-39.3; 13.9]	0.1132
**Education level**					
Primary	24.2	20.4	18.6	-23.2 [-31.6; -13.7]	<0.0001
Secondary	25.1	21.5	20.2	-19.3 [-25.8; -12.3]	<0.0001
Tertiary	16.7	11.5	12.1	-27.8 [-39.1; -14.3]	<0.0001
**Household equivalent income**					
1st quartile	28.5	25.8	22.2	-22.2 [-30.4; -13.0]	<0.0001
2nd quartile	23.9	19.8	19.2	-19.7 [-28.9; -9.3]	<0.0001
3rd quartile	23.3	17.9	17.4	-25.5 [-34.3; -15.5]	<0.0001
4th quartile	16.5	12.3	12.8	-22.6 [-33.9; -9.5]	0.0001
**Employment status**					
Employed	27.3	21.5	20.4	-25.1 [-31.0; -18.7]	<0.0001
Self-employed	25.4	21.7	19.8	-21.8 [-43.3; 7.8]	0.0604
Unemployed	47.9	36.5	41.5	-13.2 [-30.6; 8.5]	0.0633
Retired, disabled	17.6	14.2	12.1	-31.5 [-43.1; -17.5]	<0.0001
Student, apprentice	21.0	15.5	14.8	-29.5 [-43.8; -11.5]	0.0005
Other	14.3	14.7	13.8	-3.3 [-20.1; 17.0]	0.4149

Source: PSELL3/EU-SILC Survey 2005, 2007, and 2008

*p values were calculated using the Cochran-Armitage trend test.

**Table 2:** Change in the overall smoking prevalence in Luxembourg among men from 2005 to 2008.

Men	2005 (n = 3,705)	2007 (n = 3,891)	2008 (n = 3,760)	Change, 2005 to 2008, % [95% CI]	p*
**All**	27.1	21.6	20.5	-24.2 [-30.2; -17.7]	<0.0001
**Age (years)**					
16–24	30.8	25.9	25.9	-15.9 [-31.0; 2.5]	0.0264
25–34	36.1	28.0	29.8	-17.4 [-29.3; -3.6]	0.0023
35–49	28.4	23.2	20.6	-27.4 [-37.1; -16.2]	<0.0001
50–64	23.0	18.5	18.4	-20.0 [-33.9; -3.1]	0.0061
≥65	16.2	11.8	8.2	-49.5 [-63.8; -29.6]	<0.0001
**Marital status**					
Never married	31.1	25.8	26.1	-15.9 [-26.1; -4.4]	0.0016
Married	23.1	18.2	16.4	-29.0 [-37.1; -19.9]	<0.0001
Divorced/Separated	45.6	37.4	34.8	-23.7 [-38.9; -4.9]	0.0052
Widowed	22.3	14.1	12.2	-45.4 [-71.2; 3.7]	0.0204
**Education level**					
Primary	34.5	29	26.4	-23.6 [-33.7; -12.0]	<0.0001
Secondary	27.1	22.5	21.8	-19.8 [-28.2; -10.3]	<0.0001
Tertiary	18.7	12.3	12.5	-33.1 [-46.6; -16.1]	<0.0001
**Household equivalent income**					
1st quartile	35.8	29.6	27.9	-21.9 [-32.0; -10.4]	<0.0001
2nd quartile	27.8	23.9	23.0	-17.4 [-29.4; -3.3]	0.0057
3rd quartile	27.0	20.5	18.1	-33.0 [-43.6; -20.5]	<0.0001
4th quartile	18.2	13.2	14.0	-22.9 [-37.2; -5.5]	0.0019
**Employment status**					
Employed	29.7	24	22.8	-23.3 [-30.6; -15.1]	<0.0001
Self-employed	27.7	19.6	17.8	-35.6 [-56.7; -4.0]	0.0089
Unemployed	59.2	45.1	53.9	-8.9 [-27.7; 14.7]	0.1457
Retired, disabled	19.6	15.2	11.9	-39.1 [-51.4; -23.8]	<0.0001
Student, apprentice	18.4	16.3	17.4	-5.3 [-30.9; 30.0]	0.3223
Other	35.2	32.5	22.7	-35.6 [-69.5; 35.9]	0.1397

Source: PSELL3/EU-SILC Survey 2005, 2007, and 2008

*p values were calculated using the Cochran-Armitage trend test.

**Table 3:** Change in the overall smoking prevalence in Luxembourg among women from 2005 to 2008.

Women	2005 (n = 3,830)	2007 (n = 4,022)	2008 (n = 3,878)	Change, 2005 to 2008, % [95% CI]	p*
**All**	19.2	16.4	15.3	-20.2 [-27.7; -12.0]	<0.0001
**Age (years)**					
16–24	27.4	20.9	15.9	-41.9 [-54.6; -25.5]	<0.0001
25–34	17.1	15.1	17.4	1.7 [-20.1; 29.5]	0.4647
35–49	24.3	19.6	18.4	-24.1 [-35.1; -11.2]	0.0001
50–64	19.2	18.6	16.4	-14.4 [-30.5; 5.5]	0.0909
≥65	6.9	7.1	6.9	-0.6 [-32.0; 45.1]	0.4971
**Marital status**					
Never married	26.2	21.9	20.4	-22.2 [-34.0; -8.2]	0.0010
Married	15.0	12.5	11.7	-21.8 [-32.8; -9.0]	0.0004
Divorced/Separated	37.2	32.2	28.4	-23.6 [-39.1; -4.1]	0.0099
Widowed	13.1	12.7	12.4	-5.3 [-34.1; 36.1]	0.3851
**Educational level**					
Primary	16.7	14	12.9	-22.7 [-36.0; -6.1]	0.0033
Secondary	22.9	20.4	18.6	-18.7 [-28.4; -7.7]	0.0006
Tertiary	14.5	10.5	11.6	-19.8 [-38.2; 4.0]	0.0257
**Household equivalent income**					
1st quartile	21.8	22.2	17.2	-21.2 [-34.3; -5.4]	0.0153
2nd quartile	20.2	16.1	15.6	-22.6 [-36.1; -6.3]	0.0020
3rd quartile	19.9	15.5	16.7	-15.9 [-30.0; 1.2]	0.0138
4th quartile	14.7	11.3	11.3	-23.0 [-39.7; -1.6]	0.0095
**Employment status**					
Employed	23.9	18.1	17.3	-27.5 [-36.8; -16.8]	<0.0001
Self-employed	20.2	26.0	23.8	17.8 [-33.6; 108.9]	0.2532
Unemployed	31.6	28.9	24.7	-21.9 [-52.6; 28.7]	0.1771
Retired, disabled	13.8	12.5	12.3	-10.6 [-36.0; 24.8]	0.2414
Student, apprentice	23.6	14.7	12.3	-48.0 [-62.7; -27.4]	<0.0001
Other	13.8	14.2	13.5	-2.0 [-19.5; 19.3]	0.4467

Source: PSELL3/EU-SILC Survey 2005, 2007, and 2008

*p values were calculated using the Cochran-Armitage trend test.

**Fig 1:**
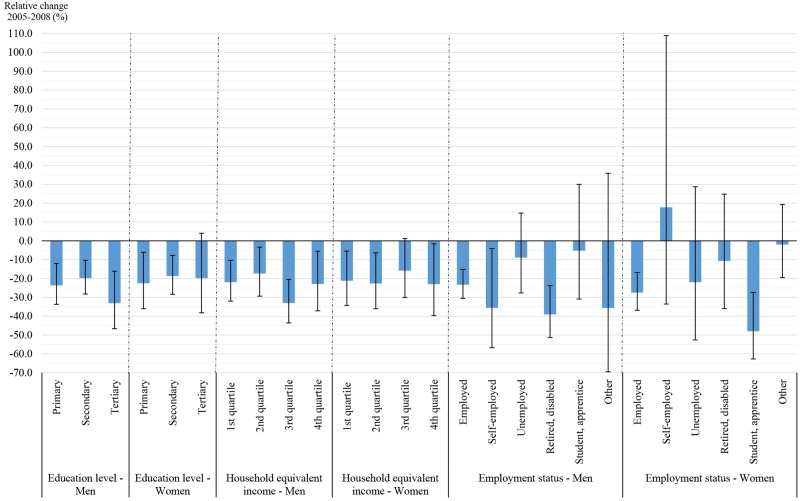
Relative change in the smoking prevalence between 2005 and 2008 in Luxembourg. Note that the vertical spikes show the 95% confidence intervals.

The relative smoking prevalence decreased for all socioeconomic groups, regardless of socioeconomic factors (except employment status for women), although to different degrees (Fig 1). Regarding education level, a sharp decrease in prevalence was observed in men with a tertiary education (-33.1%; p < 0.0001) and in women with a primary education (-22.7%; p = 0.0033). The change in smoking prevalence based on household equivalent income was greater in men in the 3^rd^ and 4^th^ quartiles (-33.0% and -22.9%, respectively) and in women in the 2^nd^ and 4^th^ quartiles (-22.6% and -23.0%, respectively). In addition, regarding employment status, the prevalence of smoking decreased in all employment categories, except for self-employed women, who experienced a 17.8% increase.

The overall decrease in smoking prevalence was not reflected across socioeconomic factors during 2005 to 2008 (Table 4). The age-adjusted smoking prevalence ratios were significantly different for each socioeconomic factor for both men and women. In men and women, the magnitude of socioeconomic inequalities in smoking, as measured using the age-adjusted prevalence ratio, remained unchanged for each socioeconomic factor. However, socioeconomic inequalities in smoking were higher for men than for women in some years.

**Table 4:** Smoking prevalence ratios based on measures of socioeconomic inequalities in education, household equivalent income, and employment status.

	Year	Men		Women	
		PRadj [95% CI]	DI [%]	PRadj [95% CI]	DI [%]
**Education level** (*Primary versus Tertiary*)	2005	2.1 [1.8; 2.5]	19.6	1.5 [1.2; 1.8]	19.1
	2007	2.7 [2.2; 3.3]	27.1	1.7 [1.3; 2.1]	24.9
	2008	2.6 [2.1; 3.2]	24.6	1.5 [1.1; 1.9]	20.5
**Household equivalent income** (*1*^*st*^ *quartile versus 4*^*th*^ *quartile*)	2005	2.0 [1.7; 2.3]	17.0	1.5 [1.2; 1.8]	11.3
	2007	2.2 [1.9; 2.7]	22.9	2.0 [1.6; 2.5]	18.5
	2008	2.0 [1.7; 2.4]	23.0	1.6 [1.2; 2.0]	12.3
**Employment status** (*Unemployed versus employed*)	2005	1.8 [1.5; 2.1]	36.7	1.2 [0.9; 1.7]	28.9
	2007	1.6 [1.3; 2.0]	39.0	1.3 [1.0; 1.8]	32.1
	2008	1.6 [1.3; 2.0]	84.8	1.3 [1.0; 1.8]	30.2

Source: PSELL3/EU-SILC Survey 2005, 2007, and 2008

PRadj, prevalence ratio between the higher and lower statuses, adjusted for age; DI, disparity index (% of total smoking prevalence); CI, confidence interval.

There were relatively small differences in the disparity index from 2005 to 2008 (19.6% to 24.6% in men and 19.1% to 20.5% in women) with respect to education level, despite a substantial increase between 2005 and 2007, followed by a decrease between 2007 and 2008 (Table 4).

Furthermore, the proportion of people who quit smoking because of the 2006 smoke-free law was only significantly different with respect to marital status, education level, and household equivalent income (Table 5).

**Table 5:** Proportion of people who quit smoking because of the 2006 smoke-free law and odds ratios for smoking cessation due to the 2006 smoke-free law in Luxembourg (in 2007).

	People who quit smoking		Model 1	Model 2	Model 3
	N	%	OR [95% CI]	OR [95% CI]	OR [95% CI]
**Age (years)**					
(16–24)	74	6.46	0.61 [0.20; 1.82]	0.52 [0.17; 1.54]	1.33 [0.32; 5.48]
(25–34)	258	10.24	1.37 [0.72; 2.62]	1.23 [0.65; 2.34]	2.11 [0.88; 5.11]
(35–49)	509	7.65	1.38 [0.79; 2.40]	1.28 [0.74; 2.20]	2.11 [0.95; 4.68]
(50–64)	522	5.3	0.93 [0.52; 1.66]	1.01 [0.57; 1.79]	1.17 [0.61; 2.25]
≥65	429	6.12	ref.	ref.	ref.
**Sex**					
Men	1146	6.89	1.07 [0.72; 1.59]	1.09 [0.73; 1.62]	1.30 [0.82; 2.05]
Women	646	6.97	ref.	ref.	ref.
**Marital status**					
Never married	263	13.5	**2.97 [1.81; 4.85]**	**3.19 [1.94; 5.24]**	**3.43 [2.07; 5.68]**
Widowed	1302	5.52	0.86 [0.35; 2.13]	1.00 [0.41; 2.44]	0.98 [0.40; 2.41]
Divorced/Separated	111	9.48	1.87 [0.94; 3.72]	1.84 [0.92; 3.66]	**2.22 [1.11; 4.45]**
Married	116	5.25	ref.	ref.	ref.
**Education level**					
Primary	450	9.44	**1.85 [1.08; 3.15]**		
Secondary	933	5.57	0.90 [0.56; 1.47]		
Tertiary	392	7.48	ref.		
**Household equivalent income**					
1st quartile	358	8.59		**2.14 [1.21; 3.80]**	
2nd quartile	456	8.64		**2.45 [1.41; 4.26]**	
3rd quartile	457	6.85		1.71 [0.97; 3.02]	
4th quartile	521	4.32		ref.	
**Employment status**					
Employed	914	7.34			ref.
Self-employed	58	3.46			0.36 [0.08; 1.52]
Unemployed	39	5.07			0.47 [0.11; 2.07]
Retired, disabled	519	6.63			1.81 [0.90; 3.63]
Student, apprentice	49	4.89			0.39 [0.07; 2.01]
Other	213	7.58			**2.18 [1.08; 4.41]**
R²			0.0498	0.0526	0.0515

Source: PSELL3/EU-SILC Survey 2005, 2007, and 2008; bold text indicates significant findings; model 1 includes education level as the socioeconomic factor; model 2 includes household equivalent income as the socioeconomic factor; and model 3 includes employment status as the socioeconomic factor.

OR, odds ratio; CI, confidence interval.

In the three logistic regression analysis models, age and sex were not associated with the probability of stopping smoking due to the 2006 smoke-free law, while marital status was (Table 5).

In model 1, smokers with a primary education had 1.85-fold greater odds (OR, 1.85; 95% confidence interval [CI], 1.08–3.15) of stopping smoking due to the 2006 smoke-free law than did those with a tertiary education. Never-married smokers had a 3-fold greater chance of quitting smoking than did married smokers. In model 2, household equivalent income was associated with smoking cessation. Smokers from the 1^st^ or 2^nd^ quartile had higher odds of stopping smoking (OR, 2.14; 95% CI, 1.21–3.80 and OR, 2.45; 95% CI, 1.41–4.26, respectively) than did those in the 4^th^ quartile. In addition, never-married smokers had 3.19-fold greater odds (OR, 3.19; 95% CI, 1.94–5.24) of stopping smoking than did married smokers. In model 3, employment status was significantly associated with smoking cessation. Smokers of an “other” profession had 2.18-fold greater odds of stopping smoking (95% CI, 1.08–4.41) than did smokers of any other employment status. Never-married smokers (OR, 3.43; 95% CI, 2.07–5.68) and divorced/separated smokers (OR, 2.22; 95% CI, 1.11–4.45) had higher odds of quitting smoking than did married smokers.

## Discussion

This study determined changes in the prevalence of smoking based on socioeconomic factors as well as the socioeconomic inequalities related to smoking cessation.

### Changes in smoking prevalence and in socioeconomic inequalities in smoking

The results of this study show that the smoking prevalence in Luxembourg is declining, as it is in almost all other developed countries that have introduced smoke-free legislation [31–34]. In the relatively short period of 2005 to 2008, the number of smokers decreased by 22.5%, or 5 percentage points. In Italy, smoking indicators were significantly higher before the smoking ban than after the ban, with an estimated reduction in the percentage of smokers of 2 points (standard error, 0.446) [31]. Another study [32] showed that a smoking ban affected the modification of individual smoking habits; on average, the smoking prevalence decreased by 1.3 percentage points.

Our results showed that socioeconomic inequalities in smoking persisted over the study period, regardless of measurement by education level, household equivalent income, or employment status.

Similarly, studies conducted in most countries have reported that differences in the prevalence of smoking among men and women based on education level persisted over time [6–8,12,19,30]. In Belgium, the results of health interview surveys conducted in 2004 and 2008 showed that the probability of being a smoker was 2.8-fold (95% CI, 2.0–4.1; 2004) and 4.6-fold (95% CI, 3.0–7.1; 2008) higher, respectively, for less educated men than for more educated men [30]. Socioeconomic inequalities were less pronounced in women (OR, 2.4; 95% CI, 1.6–3.5 in 2004 and OR, 2.9; 95% CI, 1.8–4.7 in 2008). Overall, the socioeconomic inequalities in the prevalence of smoking were more pronounced in Belgium than were those in Luxembourg.

### Socioeconomic inequalities in smoking cessation

Our results confirmed that socioeconomic inequalities are associated with smoking cessation, similar to results from other countries [8,18,20,22–25,31,34,35–37]. However, these associations depended on whether socioeconomic indicators were considered separately (models 1–3). Although education level, household equivalent income, and employment status were significantly associated with the likelihood of quitting smoking due to the smoke-free law in the separate analyses, in the model that included all three socioeconomic indicators, only household equivalent income was associated with smoking cessation (data not shown). The results were unchanged regardless of the combination of socioeconomic indicators: household equivalent income with education level, household equivalent income with employment status, or education level with employment status. In the last combination, neither education level nor employment status was associated with smoking cessation. By using separate models, we assessed the potential association of each socioeconomic indicator with smoking cessation due to the 2006 smoke-free law.

Education level in (former) smokers was associated with smoking cessation in the present study, with less educated (former) smokers (i.e., those with a primary education) being approximately 2 times more likely to quit smoking than smokers who were more educated, similar to the results of other studies [31,38]. In Italy, a greater reduction in smoking habits was observed among less educated individuals, which the authors explained by the fact that “the smoking ban is an exogenous restriction on smoking habits, which is presumed to affect more severely individuals who are not likely to quit for other (personal) reasons” [31]. However, studies conducted in other countries found conflicting results [20,22,23,25,36–37]. A recent study in Switzerland showed that a higher level of education was associated with the likelihood of successfully quitting (without relapse), both in men (OR, 1.39; *t*-statistic, 2.15) and in women (OR, 1.78; *t*-statistic, 4.49) [22]. Moreover, in Poland, a low education level was negatively associated with smoking cessation, both in men and in women [23]. In Serbia, the likelihood of stopping or quitting smoking was higher among men and more educated women (high school or university level) in the adjusted model [36]. Data from a national population survey conducted in 2001 and 2008 in the Netherlands indicated that men and women with low levels of education were less likely to quit smoking than were those who had completed graduate studies (2008: men, OR, 0.84; CI, 0.70–0.94 and women, OR, 0.56; CI, 0.47–0.67) [25].

Similar to reported findings from Romania [24], employment status in this study was associated with smoking cessation among men but not women. Men with a permanent job were more likely to quit smoking than were men who were unemployed (OR, 2.6; 95% CI, 1.32–5.09).

In the present study, (former) smokers in the two lowest household equivalent income categories had a higher probability of quitting smoking compared with those in the highest income category, which differs from the results of previous findings [22,25]. In Switzerland, men and women with the highest incomes were 1.6- and 1.5-fold more likely, respectively, to quit smoking compared to low-income participants [22]. In the Netherlands, low-income smokers had a lower probability of stopping smoking compared to that for high-income smokers, in both men (OR, 0.8; 95% CI, 0.7–1.0) and women (OR, 0.6; 95% CI, 0.5–0.7). Disadvantaged customers were less likely to quit smoking in three smoking cessation services in the UK because they did not meet the treatment guidelines [39]. An ambiguous relationship between socioeconomic status and motivation to quit has been reported previously, including mental health and stress at work [39–40]. Thus, disadvantaged smokers may be more likely to consider smoking as a way to treat or deal with other pressures and be less concerned with the health risks of smoking [40].

Using data from the Eurobarometer surveys for 11 countries, positive associations between smoking cessation rates and education and profession were detected [18]. Social inequalities in smoking cessation rates have sharply increased since the 1990s and during the 2000s, and the authors suggest that tobacco control policies implemented during the 2000s have not been able to control increasing social inequality in smoking. This trend was probably related to the fact that the measures were primarily aimed at reducing smoking among the population and at the protection of non-smokers in public places. In the US, a study by Glantz and Dinno [41] noted that “clean indoor air laws and price increases appear to benefit all socioeconomic and race/ethnic groups equally in terms of reducing smoking participation and consumption.”

Furthermore, our results showed a strong association between quitting smoking and marital status, as in other studies [32, 42].

### Limitations and strengths of this study

This study has certain limitations, including the period of observation and measurement of smoking cessation. The observation period of 1–2 years following the implementation of the smoking ban in 2006 might be too short to observe behavioural changes. The use of a questionnaire might have biased the results, because the answer to the question regarding the influence of the law on smoking cessation might have differed depending on the behaviours and beliefs of the respondent. As this study only focused on the potential impact of the 2006 smoke-free law, the proportion of smokers who ceased smoking as a result of the 2006 smoking ban is probably lower than the general smoking cessation rate. In addition, it was not possible to consider the duration of smoking cessation or the age at smoking onset. Among those who reported having quit smoking due to the smoking ban, some had definitely resumed smoking later. The risk of relapse is often higher in smokers who had stopped smoking for less than 6 months [22,43].

Moreover, data related to the intent to quit among smokers were not available; therefore, relationships with individual socioeconomic factors could not be determined. Previous studies reported that smokers with higher levels of income and/or education were more likely to have intentions of quitting or to quit smoking [10,44–45]. Although a previous study indicated a difference in smoking cessation between the sexes [23], we were unable to study sex differences owing to the relatively small number of former smokers that quit smoking after the smoke-free law. In addition, the survey from which the data were derived was not developed to evaluate smoking in the population.

However, the results of our study may have important implications in terms of policy and the development of intervention strategies to promote smoking cessation in the general population. We found that the decline in smoking was not reflected across socioeconomic status groups; smoking prevalence remains consistently higher among people of lower socioeconomic status. Policies targeted toward this group might be necessary to strengthen further reduction in both smoking prevalence and socioeconomic inequalities.

Moreover, one of the first effects of the smoke-free law in 2006 was reduced exposure to second-hand smoke by non-smokers. By reducing exposure to second-hand smoke, the smoke-free law has the potential to affect health positively at the individual and population levels [33]. The smoking ban has also had an effect on the prevalence of smoking within different social groups of the population, as the present results have shown. The largest decreases from 2005 to 2008 were observed in older people (≥65 years old, -32.3%) and the youngest people (16–24 years old, 28.2%). However, all these changes could also be due to persistent declining trends in smoking rates, other policies implemented during the same period (such as fiscal measures with tax increases for tobacco products, aid to smoking cessation program, and information and advice relating to tobacco cessation), or other unobserved factors.

Although there were differences in the magnitude of the effect based on social inequalities, our results also show a positive effect of the smoke-free law on social inequalities in smoking and smoking cessation; it was beneficial not only for people of higher socioeconomic statuses (tertiary education or 4^th^ quartile of income) but also for people of lower socioeconomic statuses (primary education or 1^st^ quartile of income). In Lithuania, similar findings were observed with respect to education level, which the authors partially attributed to the reduced acceptability of smoking in public [46]. In the Netherlands, the results from the International Tobacco Control Netherlands Survey showed no significant age-related or educational differences in successful smoking cessation after the implementation of three tobacco control interventions, although smokers aged 15–39 years were more likely to attempt to quit [34].

The reduced social acceptability of smoking was likely facilitated by the adoption of policies that banned smoking, including the provision of help for smokers to quit smoking or reduce their consumption of tobacco, such as behavioural counselling and behavioural interventions combined with pharmacotherapy [47]. Evidence suggests that psychosocial smoking cessation interventions and population-level tobacco control interventions increase the success rate of quitting [34,47].

## Conclusions

The present study showed a decreasing trend in the prevalence of smoking in Luxembourg between 2005 and 2008, with persistent socioeconomic inequalities in smoking. The proportion**s** of people who ceased smoking due to the 2006 smoke-free law were not equal among population groups; a greater proportion of people of low socioeconomic status ceased smoking. This positive result can contribute to reducing socioeconomic inequalities in smoking if the effects of the tobacco control law are sustainable. However, although the smoking ban has contributed to a reduction of such inequalities, they still persist, indicating the need for a more targeted approach of smoke-free policies directed at lower socioeconomic groups.

## Supporting Information

S1 Table Smoking prevalence in Luxembourg among men and women in 2005. (DOCX)

S2 Table Smoking prevalence in Luxembourg among men and women in 2007. (DOCX)

S3 Table Smoking prevalence in Luxembourg among men and women in 2008. (DOCX)
