# Supplementary material for: Socioeconomic Inequalities in Smoking and Smoking Cessation Due to a Smoking Ban: General Population-Based Cross-Sectional Study in Luxembourg
Source: PLoS One. 2016 Apr 21;11(4):e0153966. doi: 10.1371/journal.pone.0153966 (PMC4839754; doi:10.1371/journal.pone.0153966)
Supplement: S1 Table — (DOCX) [file pone.0153966.s001.docx]

S1 Table. Smoking prevalence in Luxembourg among men and women in 2005

|  | | **2005** | | | | | |
| --- | --- | --- | --- | --- | --- | --- | --- |
|  |  | **Men (N=3705)** | | | **Women (N=3830)** | | |
|  |  | **Smokers** | **Non-smokers** | **Chi² (p)** | **Smokers** | **Non-smokers** | **Chi² (p)** |
| **All** |  | 27.1 | 72.9 |  | 19.2 | 80.8 |  |
| **Age (years)** | 16–24 | 30.8 | 69.2 | <0.0001 | 27.4 | 72.6 | <0.0001 |
|  | 25–34 | 36.1 | 63.9 |  | 17.1 | 82.9 |  |
|  | 35–49 | 28.4 | 71.6 |  | 24.3 | 75.7 |  |
|  | 50–64 | 23.0 | 77.0 |  | 19.2 | 80.8 |  |
|  | ≥65 | 16.2 | 83.8 |  | 6.9 | 93.1 |  |
| **Marital status** | Never married | 31.1 | 68.9 | <0.0001 | 26.2 | 73.8 | <0.0001 |
|  | Married | 23.1 | 76.9 |  | 15.0 | 85.0 |  |
|  | Divorced/Separated | 45.6 | 54.4 |  | 37.2 | 62.8 |  |
|  | Widowed | 22.3 | 77.7 |  | 13.1 | 86.9 |  |
| **Educational level** | Primary | 34.5 | 65.5 | <0.0001 | 16.7 | 83.3 | <0.0001 |
|  | Secondary | 27.1 | 72.9 |  | 22.9 | 77.1 |  |
|  | Tertiary | 18.7 | 81.3 |  | 14.5 | 85.5 |  |
| **Household equivalent income** | 1st quartile | 35.8 | 64.2 | <0.0001 | 21.8 | 78.2 | 0.0008 |
|  | 2nd quartile | 27.8 | 72.2 |  | 20.2 | 79.8 |  |
|  | 3rd quartile | 27.0 | 73.0 |  | 19.9 | 80.1 |  |
|  | 4th quartile | 18.2 | 81.8 |  | 14.7 | 85.3 |  |
| **Employment status** | Employed | 29.7 | 70.3 | <0.0001 | 23.9 | 76.1 | <0.0001 |
|  | Self-employed | 27.7 | 72.3 |  | 20.2 | 79.8 |  |
|  | Unemployed | 59.2 | 40.8 |  | 31.6 | 68.4 |  |
|  | Retired, disabled | 19.6 | 80.4 |  | 13.8 | 86.2 |  |
|  | Student, apprentice | 18.4 | 81.6 |  | 23.6 | 76.4 |  |
|  | Other | 35.2 | 64.8 |  | 13.8 | 86.2 |  |

Source: PSELL3/EU-SILC Survey 2005
