# Supplementary material for: Socioeconomic Inequalities in Smoking and Smoking Cessation Due to a Smoking Ban: General Population-Based Cross-Sectional Study in Luxembourg
Source: PLoS One. 2016 Apr 21;11(4):e0153966. doi: 10.1371/journal.pone.0153966 (PMC4839754; doi:10.1371/journal.pone.0153966)
Supplement: S2 Table — (DOCX) [file pone.0153966.s002.docx]

S2 Table. Smoking prevalence in Luxembourg among men and women in 2007

|  | | **2007** | | | | | |
| --- | --- | --- | --- | --- | --- | --- | --- |
|  |  | **Men (N=3891)** | | | **Women (N=4022)** | | |
|  |  | **Smokers** | **Non-smokers** | **Chi² (p)** | **Smokers** | **Non-smokers** | **Chi² (p)** |
| **All** |  | 21.6 | 78.4 |  | 16.4 | 83.6 |  |
| **Age (years)** | 16–24 | 25.9 | 74.1 | <0.0001 | 20.9 | 79.1 | <0.0001 |
|  | 25–34 | 28.0 | 72.0 |  | 15.1 | 84.9 |  |
|  | 35–49 | 23.2 | 76.8 |  | 19.6 | 80.4 |  |
|  | 50–64 | 18.5 | 81.5 |  | 18.6 | 81.4 |  |
|  | ≥65 | 11.8 | 88.2 |  | 7.1 | 92.9 |  |
| **Marital status** | Never married | 25.8 | 74.2 | <0.0001 | 21.9 | 78.1 | <0.0001 |
|  | Married | 18.2 | 81.8 |  | 12.5 | 87.5 |  |
|  | Divorced/Separated | 37.4 | 62.6 |  | 32.2 | 67.8 |  |
|  | Widowed | 14.1 | 85.9 |  | 12.7 | 87.3 |  |
| **Educational level** | Primary | 29.0 | 71.0 | <0.0001 | 14 | 86 | <0.0001 |
|  | Secondary | 22.5 | 77.5 |  | 20.4 | 79.6 |  |
|  | Tertiary | 12.3 | 87.7 |  | 10.5 | 89.5 |  |
| **Household equivalent income** | 1st quartile | 29.6 | 70.4 | <0.0001 | 22.2 | 77.8 | <0.0001 |
|  | 2nd quartile | 23.9 | 76.1 |  | 16.1 | 83.9 |  |
|  | 3rd quartile | 20.5 | 79.5 |  | 15.5 | 84.5 |  |
|  | 4th quartile | 13.2 | 86.8 |  | 11.3 | 88.7 |  |
| **Employment status** | Employed | 24.0 | 76.0 | <0.0001 | 18.1 | 81.9 | <0.0001 |
|  | Self-employed | 19.6 | 80.4 |  | 26 | 74 |  |
|  | Unemployed | 45.1 | 54.9 |  | 28.9 | 71.1 |  |
|  | Retired, disabled | 15.2 | 84.8 |  | 12.5 | 87.5 |  |
|  | Student, apprentice | 16.3 | 83.7 |  | 14.7 | 85.3 |  |
|  | Other | 32.5 | 67.5 |  | 14.2 | 85.8 |  |

Source: PSELL3/EU-SILC Survey 2007
