# Supplementary material for: Socioeconomic Inequalities in Smoking and Smoking Cessation Due to a Smoking Ban: General Population-Based Cross-Sectional Study in Luxembourg
Source: PLoS One. 2016 Apr 21;11(4):e0153966. doi: 10.1371/journal.pone.0153966 (PMC4839754; doi:10.1371/journal.pone.0153966)
Supplement: S3 Table — (DOCX) [file pone.0153966.s003.docx]

S3 Table. Smoking prevalence in Luxembourg among men and women in 2008

|  | | **2008** | | | | | |
| --- | --- | --- | --- | --- | --- | --- | --- |
|  |  | **Men (N=3760)** | | | **Women (N=3878)** | | |
|  |  | **Smokers** | **Non-smokers** | **Chi² (p)** | **Smokers** | **Non-smokers** | **Chi² (p)** |
| **All** |  | 20.5 | 79.5 |  | 15.3 | 84.7 |  |
| **Age (years)** | 16–24 | 25.9 | 74.1 | <0.0001 | 15.9 | 84.1 | <0.0001 |
|  | 25–34 | 29.8 | 70.2 |  | 17.4 | 82.6 |  |
|  | 35–49 | 20.6 | 79.4 |  | 18.4 | 81.6 |  |
|  | 50–64 | 18.4 | 81.6 |  | 16.4 | 83.6 |  |
|  | ≥65 | 8.2 | 91.8 |  | 6.9 | 93.1 |  |
| **Marital status** | Never married | 26.1 | 73.9 | <0.0001 | 20.4 | 79.6 | <0.0001 |
|  | Married | 16.4 | 83.6 |  | 11.7 | 88.3 |  |
|  | Divorced/Separated | 34.8 | 65.2 |  | 28.4 | 71.6 |  |
|  | Widowed | 12.2 | 87.8 |  | 12.4 | 87.6 |  |
| **Educational level** | Primary | 26.4 | 73.6 | <0.0001 | 12.9 | 87.1 | <0.0001 |
|  | Secondary | 21.8 | 78.2 |  | 18.6 | 81.4 |  |
|  | Tertiary | 12.5 | 87.5 |  | 11.6 | 88.4 |  |
| **Household equivalent income** | 1st quartile | 27.9 | 72.1 | <0.0001 | 17.2 | 82.8 | 0.0017 |
|  | 2nd quartile | 23 | 77 |  | 15.6 | 84.4 |  |
|  | 3rd quartile | 18.1 | 81.9 |  | 16.7 | 83.3 |  |
|  | 4th quartile | 14 | 86 |  | 11.3 | 88.7 |  |
| **Employment status** | Employed | 22.8 | 77.2 | <0.0001 | 17.3 | 82.7 | 0.0002 |
|  | Self-employed | 17.8 | 82.2 |  | 23.8 | 76.2 |  |
|  | Unemployed | 53.9 | 46.1 |  | 24.7 | 75.3 |  |
|  | Retired, disabled | 11.9 | 88.1 |  | 12.3 | 87.7 |  |
|  | Student, apprentice | 17.4 | 82.6 |  | 12.3 | 87.7 |  |
|  | Other | 22.7 | 77.3 |  | 13.5 | 86.5 |  |

Source: PSELL3/EU-SILC Survey 2008
